# Inequalities in energy drink consumption among UK adolescents: a mixed-methods study

**DOI:** 10.1017/S1368980022002592

**Published:** 2023-03

**Authors:** Christina Vogel, Sarah Shaw, Sofia Strömmer, Sarah Crozier, Sarah Jenner, Cyrus Cooper, Janis Baird, Hazel Inskip, Mary Barker

**Affiliations:** 1 MRC Lifecourse Epidemiology Centre, University of Southampton, Southampton General Hospital, Southampton, UK; 2 Centre for Food Policy, City, University of London, London, UK; 3 NIHR Southampton Biomedical Research Centre, University of Southampton, University Hospital Southampton NHS Foundation Trust, Southampton, UK; 4 NIHR Applied Research Collaboration Wessex, Southampton Science Park, Innovation Centre, Chilworth, Southampton, UK; 5 School of Health Sciences, Faculty of Environmental and Life Sciences, University of Southampton, Southampton, UK

**Keywords:** Adolescents, Energy drinks, Diet, Policy, Inequalities

## Abstract

**Objective:**

To examine energy drink consumption among adolescents in the UK and associations with deprivation and dietary inequalities.

**Design:**

Quantitative dietary and demographic data from the National Diet and Nutrition Survey (NDNS) repeated cross-sectional survey were analysed using logistic regression models. Qualitative data from semi-structured interviews were analysed using inductive thematic analysis.

**Setting:**

UK.

**Participants:**

Quantitative data: nationally representative sample of 2587 adolescents aged 11–18 years. Qualitative data: 20 parents, 9 teachers and 28 adolescents from Hampshire, UK.

**Results:**

NDNS data showed adolescents’ consumption of energy drinks was associated with poorer dietary quality (OR 0·46 per sd; 95 % CI (0·37, 0·58); *P* < 0·001). Adolescents from more deprived areas and lower income households were more likely to consume energy drinks than those in more affluent areas and households (OR 1·40; 95 % CI (1·16, 1·69); *P* < 0·001; OR 0·98 per £1000; 95 % CI (0·96, 0·99); *P* < 0·001, respectively). Between 2008 and 2016, energy drink consumption among adolescents living in the most deprived areas increased, but decreased among those living in the most affluent neighbourhoods (*P* = 0·04). Qualitative data identified three themes. First, many adolescents drink energy drinks because of their friends and because the unbranded drinks are cheap. Second, energy drink consumption clusters with other unhealthy eating behaviours and adolescents do not know why energy drinks are unhealthy. Third, adolescents believe voluntary bans in retail outlets and schools do not work.

**Conclusions:**

This study supports the introduction of age-dependent legal restrictions on the sale of energy drinks which may help curb existing socio-economic disparities in adolescents’ energy drink intake.

Poor diet is a major contributor to the burden of non-communicable diseases and, in the UK, costs the National Health Service £6 billion annually^(1,2)^. Evidence from the annual rolling National Diet and Nutrition Survey (NDNS) shows that UK adolescents aged 11–18 years have poorer diets than other age groups^(3)^. Additionally, the Health Survey for England indicates that 23 % of adolescents aged 11–15 years are already obese; a figure that has gradually increased from 14 % in 1995^(4)^. Implementing strategies that improve adolescents’ dietary behaviours are crucial because a sub-optimal diet in adolescence affects immediate health as well as raising the risk of obesity and non-communicable diseases later in life and in the next generation^(5,6)^.

Non-alcoholic beverages are the primary source of free sugars (sugar added to food/drink or found in honey, syrup or juice) in adolescents’ diets, most of which are sugary soft drinks and energy drinks^(3)^. A survey of energy drinks for sale in the UK indicates that their average sugar content was 9·7 g/100 ml, with some drinks containing up to 16 g/100 ml^(7)^. Approximately half of the energy drinks available also have a serving size of 500 ml meaning a single bottle markedly exceeds the current dietary recommendation for free sugars which is 30 g/d for individuals aged over 11 years^(8)^.

Energy drinks are distinguishable from other soft drinks because they contain large amounts of caffeine and potentially other stimulants such as guarana, taurine and ginseng^(7)^. The energy drink survey described above identified that the average caffeine content of energy drinks is also high at 31·6 mg/100 ml (±0·8), equating to 158 mg of caffeine in a 500 ml bottle^(8)^. The current European caffeine recommendations specify a daily allowance of 3 mg of caffeine/kg body weight^(9)^. A single serving of these drinks therefore surpasses this recommendation for adolescents with a body weight below 53 kg. Among adolescents energy drink consumption has been linked to several physical symptoms including headaches, stomach aches, hyperactivity and insomnia; these symptoms largely relate to the high caffeine and sugar content of energy drinks^(10)^.

Energy drink sales have grown substantially over the past decade with current UK sales estimated at 680 million l/year^(11)^. Alarmingly, a European Food Safety Authority report indicated that young people aged 10–17 years are the greatest energy drink consumers^(12)^. The report’s statistics indicate that British adolescents consumed the greatest quantity of energy drinks of all participating European countries, consuming over a litre a month more than the European average of 2 l/month. The report also showed that more older adolescents (73 %) and boys (74 %) reported consuming energy drinks than younger adolescents (55 %) and girls (63 %).

Increased awareness of the potential dangers that energy drinks pose to young people’s health has led several major retailers to impose voluntary bans on the sale of energy drinks to minors under 16 years^(13)^; many UK schools have also introduced voluntary bans to prevent students drinking them on school premises^(14)^. In 2018, the UK Government undertook a consultation on their proposal to introduce legislation to ban the sale of energy drinks to minors; they proposed this would create consistency across retailers and protect young people’s health^(15)^. The House of Commons Science and Technology Committee released an advisory report at the end of 2018 outlining their interpretation of the evidence and recommendations to government. They concluded that there was insufficient evidence to warrant introducing a ban on selling energy drinks to children^(16)^. The Committee’s report acknowledged that energy drinks were consumed disproportionately by disadvantaged groups but noted that evidence of this trend worsening over time or undermining educational or health outcomes was needed for action to be taken. Additionally, insufficient evidence about the impact of voluntary bans was highlighted, with recognition that qualitative evidence from teachers and parents could indicate societal concerns that would provide legitimacy for a statutory ban. Contrary to the Committee’s recommendations, the ‘Advancing our Health: prevention in the 2020’s’ green paper, released in 2019, announced that the UK Government intended to introduce a ban on the sale of energy drinks to individuals aged under 16 years^(17)^. The basis for this ban was largely founded on the rationale that reductions in energy drink consumption would decrease calorie intake and improve diet, thereby helping to lower obesity rates. Providing scientific evidence of these associations would further support government intervention and is necessary because the exact details of this policy are yet to be published.

To address existing evidence gaps and provide robust scientific evidence to inform policy change, we conducted a mixed-methods study combining data from a national dietary dataset with qualitative data from interviews with adolescents, parents and teachers. The specific aims were:

1. To determine the prevalence of energy drink consumption among adolescents in the UK and assess how consumption varies by gender and age group.
2. To examine associations between energy drink consumption among adolescents in the UK and deprivation and dietary inequalities.
3. To explore teachers’, parents’ and adolescents’ perceptions of adolescent energy drink consumption and the effectiveness of current voluntary energy drink restrictions in schools and supermarkets.

## Method

### Study design and setting

This study adopted a mixed-methods study design. Quantitative data were used to address the first two research aims and qualitative data used to address the third aim. Qualitative and quantitative datasets were then combined to corroborate findings and expand the breadth and depth of interpretation. Quantitative data were taken from the NDNS rolling programme, a repeated cross-sectional survey conducted with a representative sample of the national population. Each year the NDNS programme recruits approximately 500 adults and 500 children aged over 18 months from randomly selected households across the UK. Participants (or their parents) are asked to complete a face-to-face questionnaire about household and individual demographics as well as an estimated food diary (food/drink is not weighed to reduce participant burden).

Qualitative data were collected as part of the development work for the Engaging Adolescents in CHanging Behaviour (EACH-B) study, a multi-component intervention to support adolescent diet and physical activity^(18)^. This formative work was conducted in community settings in Hampshire, UK. All elements of this study were conducted according to the Declaration of Helsinki and data protection regulations and were approved by the University of Southampton, Faculty of Medicine Ethics Committee (ethics approval number 30054).

### Quantitative data: National Diet and Nutrition Survey

Dietary intake data were derived from food diaries. Participants recorded details of all foods and drinks consumed on up to four consecutive days, with estimated portion sizes, brand names or ingredients for homemade meals. Trained NDNS coders classified the items in the diaries into 154 food groups and assigned energy values (kcals). Detailed descriptions of the design and methodology can be found elsewhere^(19)^. Our analyses were performed on 2587 adolescents aged 11–18 years from the combined survey waves 1–8, from 2008 to 2016.

Frequency of energy drink consumption was calculated for each participant, adjusting for the number of diary days completed. As energy drinks are not categorised into their own NDNS food group, the names of all items categorised in the ‘Soft drink, not diet’ and ‘Soft drinks, diet’ categories were extracted and reviewed. Energy drinks were defined as drinks (excluding tea and coffee) containing over 150 mg of caffeine (but those with low caffeine (> 100 and < 150 mg/l) were excluded) in accordance with European Union labelling regulations for high-caffeine products requiring warning labels for children^(16)^. Energy drinks with low or no sugar were included.

Total daily energy intake was calculated for each participant by summing the energy for all the food and drink items consumed and averaging over the number of diary days. A diet quality score was derived for each participant using NDNS data using a published methodology^(20)^. Diet quality scores were generated using principal component analysis on 139 food groups; vitamins, minerals and artificial sweetener groups were removed. principal component analysis is a commonly used method for generating dietary patterns^(21)^. The first component of the principal component analysis explained the greatest variance in the dietary data and represented a diet consistent with UK dietary recommendation: higher consumption of fruit, vegetables, wholegrains and lower intake of sugar-sweetened beverages, chips and processed meats. The principal component analysis allocated coefficients to each food group to quantify their contribution to the overall component. The coefficients and reported frequencies of consumption were used to calculate a dietary quality score for each participant. To facilitate interpretation of the results, dietary quality scores were standardised to a mean of zero and a SD of one with higher scores representing better quality diets; dietary scores have been validated against fourteen nutritional biomarkers, including serum folate, homocysteine, total carotenoids and vitamins B_12_, C and D^(20)^.

An equivalised household income variable was developed using total household income reported by the main food provider, adjusted for household size and demands. Index of multiple deprivation (IMD), the official measure of relative deprivation for small areas in England, was calculated for each participant based on the household postcode^(22)^. IMD scores were divided into quintiles and used to determine the neighbourhood deprivation for each household. IMD was not recorded in the NDNS for waves 5 and 6 due to changes in the study protocol. BMI *Z*-scores were created to adjust for age and sex^(23)^ and categorised according to cut-offs defined from nationally representative surveys with adolescents.

### Qualitative data: interviews with adolescents, parent and teachers

Semi-structured interviews were conducted with parents, teachers and adolescents to learn about adolescents’ daily food and physical activity habits and what could support healthier choices. Interviews were conducted with an additional sample of adolescents to explore energy drink consumption in more depth. All interviews were conducted using semi-structured topic guides distinct for each participant group (available on request). Participants were not shown the questions in advance.

Participants were recruited in 2018 from a secondary school, a community youth club and at a hospital open day. Adolescents were interviewed at their school or youth club. The school was a non-selective mixed secondary school where above-average numbers of students were eligible for free school meals (36·7 %), compared to the national average (28·6 %). The youth club targeted adolescents from disadvantaged backgrounds with low school attendance. Teachers at the school were interviewed during working hours. Parents were interviewed at their workplace or home by telephone or in person at a hospital evening event for parents. Adolescents and teachers were interviewed in either pairs or groups of three to six participants; parents were interviewed individually or in groups containing three participants. All face-to-face interviews were conducted by one researcher (S.St., S.Sh. or S.J.) with an observer present who took notes (S.St., S.Sh., S.J., D.W., D.P.N. or T.M.); telephone interviews were conducted by one researcher (S.St., S.Sh. or S.J.) and with a single participant. Interviews were transcribed verbatim and pooled together into a single NVivo project (QSR International Pty Ltd., 2018: version 12). Participants did not comment on their transcripts.

### Quantitative data analyses

Summary statistics were used to describe NDNS sample characteristics: mean (sd) for normally distributed continuous variables and median (IQR) for non-normally distributed continuous variables. Frequencies are quoted for binary variables. Frequency of energy drink consumption was calculated per participant, adjusting for the fact that 1·8 % of diaries were completed for only three of the 4 d (98·2 % of participants completed 4 d diaries). Energy drink consumption was highly skewed, with 93·0 % of children reporting consuming no energy drinks in their food diaries; these data were therefore analysed as a dichotomous variable (consumer *v*. non-consumer). To assess differences in energy drink consumption according to age, gender and neighbourhood deprivation, the proportions of energy drink consumers were calculated across categories of demographic variables. Logistic regression models were fitted with energy drink consumption as the outcome to describe the effects of demographic variables on energy drink consumption. A logistic regression model was fitted with IMD, year of study and the interaction between the two as predictor variables; the interaction term describes whether the effects of IMD differ as the year of study increases. To assess whether energy drink intake related to dietary quality, diet scores were divided into tertiles describing poorer, medium and higher quality diets. Age was divided into two groups, 11–15 and 16–18 years because most adolescents commence secondary school at age 11 and college at age 16. Household income was divided into two categories (<£27 000 and ≥£27 000) which is reflective of the UK median household income in 2012, the midpoint for the data time period^(24)^. Daily energy intake was divided into four groups (<1400 kcals, 1400 kcal–<1700 kcal, 1700–< 2000 kcal, ≥2000 kcal). Changes in diet quality score are interpreted in terms of the original foods consumed by calculating the equivalent change on the original scale to the change from the median on the Fisher–Yates transformed scale.

Weights were provided in the NDNS dataset to adjust for the under-representation of children in households with more than one child (only one adult and up to one child were recruited per household) and for the cluster identifier (small geographic postcode sectors randomly selected from across the UK from which addresses were randomly selected). The weights were rescaled to reflect different sample sizes in different waves so all data could be combined. Weighted analyses are presented throughout.

### Qualitative data analysis

NVivo queries were used to extract the broad context of any references to ‘energy drinks’, and popular brands in the UK e.g. ‘Red Bull’, ‘Monster’ and ‘Rockstar’. ‘Lucozade’ was also included because brands with lower caffeine levels are colloquially called energy drinks^(16)^. Quotes were analysed using conventional content analysis following established guidelines^(25)^. Initial codes were developed by C.V. and S.J. by creating ‘nodes’ in NVivo as new topics arose. After all transcripts had been coded, ‘nodes’ were refined with input from S.Sh., S.St. and M.B. and organised into themes and sub-themes. This approach is aligned with a relativist ontological and subjective epistemic position, which purports that reality is a matter of individual perspective and based on personal experience and insight^(26)^. To ensure the interpretation was an accurate representation of interviewees’ views and data analysis decisions were transparent, a rigorous process was adopted in which data were double-coded by pairs of the researchers, and disagreements were resolved in team discussions throughout the coding process. The five researchers involved in the qualitative analysis were all women, their expertise were in nutrition and/or psychology and their ages varied from young adult to middle age.

## Results

### Participant characteristics: quantitative data

The quantitative analysis sample comprised all 2587 adolescents, 1305 girls and 1282 boys (aged 11–18 years) in waves 1–8 (2008–2016) of the NDNS dataset (Table 1). The majority were aged 11–15 years (61 %), of white ethnicity (89 %) and lived in households with <£27 000/year (66 %). The distribution of BMI *Z*-score was similar by age categories, such that 31 % of those aged 11–15 were classified as overweight or obese, compared to 27 % of those aged 16–18.

**Table 1:** Characteristics of NDNS sample (*n* 2587)

Characteristics	Summary statistics	
	*n*	%
Gender		
Girls	1305	50·4
Boys	1282	49·6
Age (years)		
11–15 years	1572	60·8
16–18 years	1015	39·2
Ethnicity		
White	2309	89·3
Other	276	10·7
Equivalised household income (£)		
<27 000	1524	65·7
≥27 000	797	34·3
IMD		
Two least deprived quintiles	666	40·0
Three most deprived quintiles	999	60·0
BMI (kg/m^2^)		
Thin	149	6·0
Healthy weight	1613	64·7
Overweigh	506	20·3
Obese	225	9·0
Completed four diary days		
Yes	2541	98·2
No	46	1·8
Energy drink consumer		
Yes	182	7·0
No	2405	93·0
	Median	IQR
Energy intake (kcal/d)	1711	1401, 2052

IMD, index of multiple deprivation; NDNS, National Diet and Nutrition Survey.

### Participant characteristics: qualitative data

Of the fifty-seven interviews conducted, twenty-eight were with adolescents (*n* 74), twenty with parents (*n* 24) and nine with teachers (*n* 15) (Table 2). Demographic data were not collected from two adolescent group interviews (∼25 %) due to time restrictions. Of the adolescent participants who provided demographic data (*n* 55), most were aged 13–14 years (95 %) and of white ethnicity (98 %); fewer than half were girls (42 %). The majority of parents and teachers were women (100 % and 80 %, respectively) and of white ethnicity (96 % and 87 %, respectively); most parents were aged 40–49 years (71 %), while almost three-quarters of teachers were aged 20–39 years (73 %).

**Table 2:** Characteristics of qualitative sample

Characteristic	Summary statistic					
	Adolescents *n* 74		Parents *n* 24		Teachers *n* 15	
	*n*	%	*n*	%	*n*	%
Age						
13 years	26	35·1	–	–	–	–
14 years	26	35·1	–	–	–	–
15 years	1	1·4	–	–	–	–
16 years	1	1·4	–	–	–	–
17 years	1	1·4	–	–	–	–
20–29	–	–	–	–	6	40
30–39	–	–	2	8	5	33·3
40–49	–	–	17	71	4	26·7
50+	–	–	5	21	0	0
Missing	19	25·7	–	–	–	–
Gender						
Girl/women	23	31·1	24	100	12	80·0
Boy/man	31	41·9	–	–	3	20·0
Other	1	1·4	–	–	–	–
Missing	19	25·7	–	–	–	–
Ethnicity						
White	54	73·0	23	96	13	86·7
Other	1	1·4	1	4	2	13
Missing	19	25·7	–	–	–	

### Aim 1: prevalence of energy drink consumption among adolescents in the UK

The NDNS data showed that 7·0 % of adolescents consumed at least one energy drink in a 4 d period. Older adolescents were more likely to consume energy drinks than younger adolescents (Table 3). A 1-year increase in age was associated with a 21 % increase in the likelihood of energy drink consumption (OR 1·21; 95 % CI (1·12, 1·31); *P* < 0·001). This trend of increased energy drink consumption through adolescence did not decline over time, despite the known increase in public awareness of safety concerns regarding energy drinks (*P* = 0·50). No difference was observed between the proportion of girls and boys consuming energy drinks (*P* = 0·81).

**Table 3:** Associations of energy drink consumption status with participant demographics for 11–18 year olds

	Consuming energy drinks (*n*)	Total (*n*)	Weighted proportion consuming energy drinks	OR	95 % CI	*P*-value
Age (years)						
11–15	93	1572	4·9	–	–	–
16–18	89	1015	8·3	–	–	–
Trend per year	–	–	–	1·21	1·12, 1·31^*^	< 0·001
Gender						
Boys	100	1282	6·1	Baseline	–	–
Girls	82	1305	6·4	1·05	0·69, 1·61	0·81
IMD						
1 (least deprived)	17	347	3·5	–	–	–
2	12	319	3·0	–	–	–
3	16	318	4·6	–	–	–
4	20	342	6·8	–	–	–
5 (most deprived)	32	339	10·4	–	–	–
Trend	–	–	–	1·40	1·16, 1·69^†^	< 0·001
Equivalised household income						
< £27 000	128	1524	7·6	–	–	–
≥ £27 000	36	797	3·7	–	–	–
Trend per £1000	–	–	–	0·98	0·96, 0·99^‡^	< 0·001
Diet quality score						
Low	102	863	10·7	–	–	–
Medium	59	862	6·6	–	–	–
High	21	862	2·3	–	–	–
Trend	–	–	–	0·46	0·37, 0·58^§^	< 0·001
Energy intake (kcal)						
< 1400	32	645	5·6	–	–	–
≥ 1400 and < 1700	42	635	4·3	–	–	–
≥ 1700 and < 2000	41	594	6·6	–	–	–
≥ 2000	67	713	8·2	–		–
Trend per 100 kcal	–	–	–	1·04	1·01, 1·08^‖^	0·02
BMI (kg/m^2^)						
Thin	15	149	9·4	–	–	–
Healthy weight	116	1613	5·6	–	–	–
Overweight	26	506	5·3	–	–	–
Obese	20	225	10·1	–	–	–
Trend per sd BMI *Z*-score	–	–	–	1·09	0·90, 1·31^¶^	0·40

* Age modelled as a continuous variable in years.

† Index of multiple deprivation (IMD) quintile modelled as a continuous variable.

‡ Income modelled as a continuous variable per £1000.

§ Diet quality score modelled as a continuous variable (sd units).

‖ Energy intake modelled as continuous variable per 100 kcal.

¶ BMI modelled as a continuous variable.

### Aim 2: associations between energy drink consumption and deprivation and dietary inequalities

**Fig. 1:**
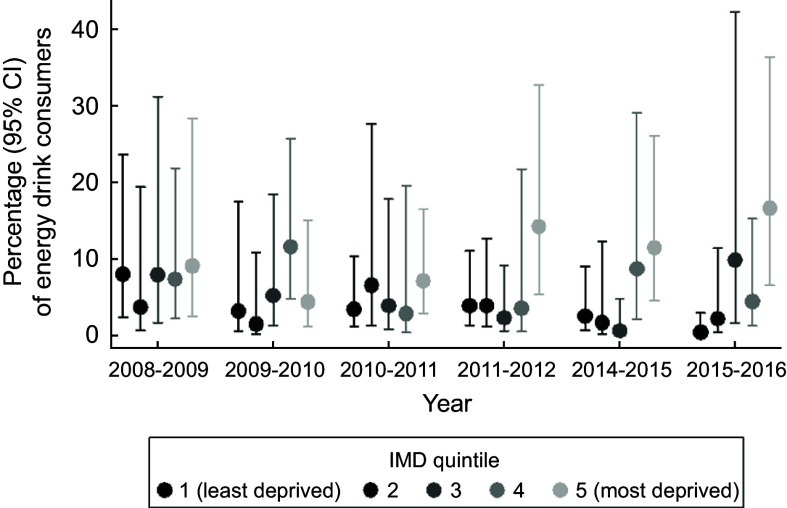
Association between IMD and energy drink consumption over time among 11–18 year olds. Note: Index of multiple deprivation (IMD) was not collected in waves 5 (2012–2013) and 6 (2013–2014)

Adolescents in more deprived areas consumed energy drinks more frequently than those in more affluent areas (Table 3). A one quintile increase in IMD was associated with a 40 % increased likelihood of consuming energy drinks (OR 1·40; 95 % CI (1·16, 1·69); *P* < 0·001). Similarly, adolescents from lower annual income households were more likely to consume energy drinks compared to those from higher annual income households (Table 3). A £1000 increase in household income was associated with being 2 % less likely to consume energy drinks (OR 0·98; 95 % CI (0·96, 0·99); *P* < 0·001). Between 2008 and 2016, energy drink consumption among adolescents living in the most deprived areas increased, whilst consumption among those living in the most affluent neighbourhoods decreased (*P* for interaction of year and IMD = 0·04) (Fig. 1).

Adolescents’ consumption of energy drinks was also associated with poorer dietary quality (Table 3). A 1 sd increase in dietary quality score was associated with being 54 % less likely to consume energy drinks (OR 0·46; 95 % CI (0·37, 0·58); *P* < 0·001). Changes in diet quality scores can be achieved in many ways; an illustration of 1 sd higher dietary quality score is consuming seven additional portions of nuts and seeds and six additional portions of salad and other raw vegetables/week, plus six fewer portions of chips and six fewer portions of sugar-sweetened carbonated-drinks/week. A 100 kcal increase in daily energy intake was also associated with 4 % increased likelihood of consuming energy drinks (OR 1·04; 95 % CI (1·01, 1·08)). Additionally, a 1 SD increase in BMI score was associated with 9 % increased likelihood of consuming energy drinks (OR 1·09; 95 % CI (0·90, 1·31); *P* = 0·40).

### Aim 3: to explore teachers’, parents’ and adolescents’ perceptions of adolescent energy drink consumption and the effectiveness of current voluntary energy drink restrictions in schools and supermarkets

Three dominant themes were identified from the qualitative interviews, which are summarised below along with illustrative quotes.

#### Theme 1: a lot of young people drink energy drinks – friends and price are key reasons why

Many of the adolescents interviewed mentioned consuming energy drinks weekly or monthly, and a number reported more frequent consumption. For several adolescents, energy drinks were part of their daily routine:

‘I like Rockstar. It’s the only thing I drink’ (Adolescent interview, school 13)

‘I buys one [energy drink] every time I gets chip shop though, and I gets chip shop like- every day.’ (Adolescent interview, youth club 2)

Adolescents sometimes struggled to say ‘no’ to energy drinks when they were offered them by their peers, but some had made a conscious decision to reduce their consumption after learning what the drinks contained. These adolescents expressed confusion when they saw their parents or other adults drinking them:

‘If someone buys me one, I have a sip of it, but then give it away… I know what’s in them now’ (Adolescent interview, school 13)

‘My dad drinks like a big can of energy drink, like Rockstar, he drinks that…My mum’s cousin was drinking energy drinks literally all the time.’ (Adolescent interview, youth club 7)

Some adolescents described the high price and taste of energy drinks as a deterrent; however, many reported enjoying the taste and reported consuming the unbranded energy drinks because of their low cost:

‘I’d rather just go buy a can of coke for that [price]. And it’s tastier.’ (Adolescent interview, youth club 3)

The cheap 30p ones [energy drinks]……better than paying like one pound forty for Red Bull.” (Adolescent interview, youth club 1)

Parents and teachers were also aware that many adolescents consume energy drinks regularly. Teachers raised concerns about the social desirability of energy drinks among adolescents and most acknowledged that energy drink consumption had become an accepted norm among their students and conformed to the social pressures:

‘You can see what they’ve been buying on this App. And these fruit drinks, which are energy drinks, he bought four in 1 day’ (Parent interview 1)

‘There’s definitely a social pressure. If their friends [say], ‘oh I have five energy drinks a day’, there is just a constant pressure. I think they’re at that point where they’re thinking about who they are and who they want to be’ (Teacher interview 3)

#### Theme 2: energy drinks are not good for health and cluster with unhealthy diets

Adolescents largely recognised that energy drinks are not good for their health and some had experienced negative physical side effects from drinking them. Many, however, were confused over exactly why energy drinks are deemed so unhealthy:

‘I can’t drink them much ‘cause it makes me have really bad belly ache, and makes my chest feel even worse.’ (Adolescent interview, youth club 6)

I: ‘You said they did something at school about it [energy drinks]?’

P: ‘Yeah, I dunno they just said sugar.’ (Adolescent interview, youth club 1)

Teachers described having witnessed the harmful physical and behavioural effects energy drinks have had on their students. They also said that energy drink consumption was often accompanied by eating unhealthy foods or under eating; some teachers expressed alarm at how this affected students’ school attendance or ability to learn:

‘I had a GCSE student [who] was making himself ill by drinking these energy drinks, he’d have one every morning but not eat anything. About mid-day he’d feel really ill, and he’d go home. He’d have stomach cramps, a headache.’ (Teacher interview 2)

‘Their breakfast is a packet of crisps and an energy drink’ (Teacher interview 4)

#### Theme 3: voluntary bans do not work

Adolescents described how the voluntary bans in place in food retail outlets did not prevent them from being sold energy drinks, particularly in smaller, convenience stores. Some adolescents did not think this was right but were also pleased they could buy energy drinks if they wanted them, indicating a conflict between societal and individual needs:

‘They [brought] out that law like a couple of years ago didn’t they, that [shops] weren’t allowed to sell energy drinks and they still do. They still sell them to me, and I’m only fourteen’ (Adolescent interview, youth club 3)

‘Even my little sister drinks them – she’s eleven … my eleven year old sister would be able to go in there [local shop], get a can of energy drink.’ (Girl, ED interview 2)

Adolescents also felt that profits were more important to some businesses than following the voluntary bans to sell energy drinks to minors. These sorts of statements were said with disdain towards the shop owners indicating disapproval of this approach:

‘Shops just don’t care, as long as they’re making their money.’ (Adolescent interview, youth club 7)

Teachers also reported that voluntary bans in schools had limited effectiveness on reducing energy drink intake. Teachers were aware that students smuggled energy drinks into school and acknowledged that enforcing the school bans were not always easy:

‘I know quite a few of them still have Lucozade though and Red Bull…. They do, they hide it in their bags very well.’ [Teacher interview 13]

Some adolescents felt that if energy drinks are detrimental to their health, access to them in stores should be restricted so they were more difficult to buy. Other adolescents, however, were adamant that they would find ways around stricter sales restrictions:

‘But then energy drinks, if they’re that bad and they’ve gotta have ID then surely they shouldn’t be in the fridge, they should be behind the tills with all the alcohol.’ (Adolescent interview, youth club 7)

‘We’d get people to get them for us … and I’d get it from the shop myself ‘cose I know all the shops round here and they all love me.’ (Adolescent interview, youth club 6)

## Discussion

### Main findings

This mixed-methods study used a nationally representative dietary dataset (*n* 2587) to characterise inequalities in energy drink consumption and semi-structured interviews with a large sample of adolescents, parents and teachers (*n* 113) to provide deeper insight into adolescent energy drink intake and effectiveness of its current regulation. The findings showed that the overall prevalence of adolescents’ energy drink consumption over a 4-d period was 7·0 %, and that consumption rates were higher among older adolescents, regardless of gender (Aim 1). Additionally, this trend of greater energy drink consumption with age did not decline over time. This study demonstrated clear associations between adolescent energy drink intake and markers of socio-economic deprivation and dietary inequalities (Aim 2). Adolescents living in more deprived areas and from lower income households were considerably more likely to consume energy drinks than those from more affluent areas and households, and higher energy drink consumption was associated with poorer dietary quality, higher energy intake and greater body mass. Worryingly, inequalities in energy drink consumption by area deprivation increased over the 8 year timeframe of the quantitative dataset, with rates increasing among those from the most deprived areas and decreasing among those most affluent.

Three themes were identified from the interviews with adolescents, parents and teachers (Aim 3). First, many adolescents who drink energy drinks do so because of their friends and because the unbranded drinks are cheap. Second, energy drink consumption clusters with other unhealthy eating behaviours and the harmful physical effects of energy drinks have been witnessed by teachers and some parents; yet many adolescents do not know exactly why energy drinks are unhealthy (Aim 3). Third, participants generally felt that voluntary bans in retail outlets, particularly smaller stores, and in schools do not work; many favoured the introduction of legal restrictions on selling energy drinks to minors but some felt they could find ways to circumvent tougher restrictions.

### Comparison with previous research

The prevalence estimate of adolescent energy drink consumption from this study seems lower than previous research from a similar point in time, including findings from the World Health Organisations’ European Health Behaviour in School-aged Children study which indicates that energy drink consumption rates across countries range from 9 to 24 %^(27-30)^. Such differences in prevalence rates may be due to variations in data collection methods. These previous studies asked about energy drink intake within the past week and reported prevalence rates of 15, 21 and 24 % for adolescent consumption at least once a week, and 9 % prevalence for consumption 2–4 times a week. The current study used food diaries of up to 4 d. It is therefore likely that due to this short time frame, our findings offer a more conservative estimate of energy drink consumption compared with other studies and indicate the prevalence of very frequent, or daily, energy drink intake among adolescents in the UK.

Teachers and parents perceived that energy drinks were associated with a specific social identity which fuelled their popularity among adolescents. Energy drink consumption has previously been linked to group membership and social identity among young people^(31)^. In this study, social status acquired from energy drink consumption was not expressed explicitly by adolescent participants and it is unclear how aware they were of their behaviour being influenced by cultural norms. Some adolescents, however, did mention feeling pressured to partake when energy drinks were being circulated by their peers. Adolescents are known to value social acceptance and group membership, but simultaneously strive for autonomy^(32)^. These somewhat conflicting determinants of behaviour may help to explain a reluctance in revealing or understanding the true motives for their energy drink consumption.

Internationally, research has shown that energy drinks are consumed more frequently by older adolescents and by those from more disadvantaged backgrounds^(28,30,33,34)^. Our findings align with this previous work, showing that each additional year of age increased the likelihood of consuming energy drinks by 21 %, with highest rates among 17 and 18 year olds. This pattern likely reflects the growing levels of independence over food choices that adolescents acquire with age, and challenges the UK Government’s proposal to prohibit the sale of energy drinks to those under 16 years of age. Applying the cut-point at 18 years of age would be more consistent with the evidence on energy drink intake and could help protect older adolescents from more disadvantaged backgrounds who appear to be particularly vulnerable to the regular intake of energy drinks.

A disturbing pattern of increasing inequalities in energy drink consumption was revealed in our study, whereby intakes among adolescents from the most deprived communities increased over an 8-year period while intakes among those from affluent communities decreased. Clustering of unhealthy behaviours among energy drink consumers was also apparent in both our quantitative and qualitative data results, showing that energy drink consumers had poorer quality diets, higher daily energy intake and larger BMI. Previous research has shown that multiple unhealthy behaviours cluster among young people from disadvantaged backgrounds, particularly low fruit and vegetable intake and high tobacco and alcohol use, as well as low fruit and vegetable intake and low physical activity levels coupled with high sedentary behaviour and high sugary drinks intake^(35,36)^. In our study, each additional sd increase in BMI was associated with 9 % greater likelihood of adolescents’ consuming energy drinks. Although not statistically significant, this may suggest the simultaneous occurrence of health-compromising behaviours that could accentuate the risk of non-communicable diseases among these young people. This higher risk has implications for themselves, their future off-spring and society. Interventions to reverse entrenched inequalities are likely to be most successful if they target multiple risk behaviours and address social and environmental drivers^(37)^.

### Implications for policy

The findings from this study support the UK Government’s plans to introduce legislation to end the sale of energy drinks to minors^(17)^; it suggests that voluntary bans in large supermarket chains and schools are not implemented effectively and are undermined by smaller convenience stores who continue to sell these products to adolescents. More deprived neighbourhoods have higher concentrations of convenience stores and poorer in-store environments^(38,39)^. Such unhealthy environmental exposures have been shown to exacerbate existing dietary inequalities^(40-42)^ and may be contributing to the increasing disparity in energy drink consumption between adolescents from more deprived and more affluent areas; legislation may therefore help to address inequalities.

Importantly, this study highlights that the proposed legislation would miss the opportunity to reduce consumption among the highest energy drink-consuming adolescents; those aged 16–18 years. The limit of 16 years may be challenging to implement and easier for younger adolescents to work around. Well-established age restrictions on the sale of tobacco and alcohol to those aged under 18 years already exist in the UK. Aligning the limits on the sale of energy drinks with these established legislations would provide a clear message to the public that these drinks are not suitable for adolescents, as well as facilitating consistent enforcement across all retail premises.

For the proposed legislation to be maximally effective additional actions could be considered by policymakers, including minimum pricing of energy drinks and positioning them in restricted areas of retail outlets. The cheap price of own-brand energy drinks was identified as a key determinant of their consumption by adolescents, particularly those from disadvantaged backgrounds, in this and previous research^(16)^. Introducing a minimum pricing of energy drinks could successfully limit intake in a similar way that the introduction of minimum alcohol pricing in Scotland showed immediate impact, reducing alcohol purchases among lower income and higher alcohol-purchasing households^(43)^. Additionally, adolescents interviewed in this study suggested that placing energy drinks behind counters with tobacco and some alcohol products would clearly indicate a health warning and make them less accessible to young people. There is increasing evidence that product placement influences purchasing patterns and could be used to support health behaviours, including among adolescents^(44,45)^.

A communications campaigns about the harmful effects of energy drinks may also be warranted. While there is good evidence illustrating the harmful and unpleasant physiological effects of energy drinks^(46)^, adolescents taking part in this study did not truly understand what made energy drinks so dangerous. Recent evidence indicates the harmful physiological effects of energy drinks on the cardiovascular system occur independently from caffeine, possibly caused by the additional energy-boosting substances such as taurine, guarana and sugar^(47)^. Overuse of energy drinks has caused sudden cardiac death, poor mental health and hinders academic performance^(48)^; these risks need to be appropriately communicated to young people and their families. Future research could: (i) test how labelling strategies, such as warning labels, may help to inform adolescents about the dangers of energy drinks^(49)^ and (ii) co-create the design of communication strategies that align with adolescents’ values of autonomy and fun while informing them of healthier alternatives to energy drinks^(50)^.

### Strengths and limitations

The use of mixed-methods is a strength of this study because it enables a more nuanced understanding of how energy drinks fit into the lives of young people in the UK. The quantitative analyses used a nationally representative dataset that is representative of, and generalisable to, the UK adolescent population. Self-report dietary assessment methods have been shown to be prone to under-reporting and thus reporting bias may have been possible, particularly among adolescents from more advantaged backgrounds^(51)^. The qualitative data included views from a range of population groups – adolescents, parents and teachers – different genders and individuals living in more disadvantaged areas. The interviews were conducted in pairs or groups which may have affected the responses received due to the dynamics between participants. For example, very close friends being interviewed as a pair may have given more detailed responses than a larger group interview with members from different friendship groups or different genders. Offering only large or smaller groups may have limited the scope of information received from participants. A methodological consideration is that the qualitative data were collected from a single southern county in the UK and that most participants were white. Unlike the quantitative data, the qualitative sample is therefore not representative of adolescents across England; however, recruitment strategies targeting lower income youth clubs and schools aimed to improve representation across the socio-economic spectrum. Interviews with more diverse groups of adolescents from a different area may have produced different information.

## Conclusions

This study supports the introduction of legal restrictions on the sale of energy drinks to minors but indicates that prohibiting energy drink sales to those under the age of 16 years would miss the opportunity to reduce consumption among the highest consumers, those aged 16–18 years from disadvantaged backgrounds. Such restrictions would level the playing field between retailers and may be maximumly effective if coupled with minimum-pricing strategies, placement restrictions and a communications campaign detailing their harmful effects.
